# Relationship between Aging and Ketogenic Diet: A Bibliometric Analysis (1995-2025)

**DOI:** 10.2174/0118715303376457250617154223

**Published:** 2025-06-30

**Authors:** Tugba Elgun, Ayşe Akgul Isik, Enver Ciraci, Gul Ipek Gundogan, Kaan Ziksahna, Halil Ibrahim Arslan

**Affiliations:** 1 Department of Medical Biology, Faculty of Medicine, Biruni University, Istanbul, Turkey;; 2 Biruni University Research Center (B@MER), Biruni University, 34015, Istanbul,Turkey;; 3 Medical Laboratory TechniquesProgram, Vocational School of Health Services, Biruni University, Istanbul, Turkey;; 4 Department of Biochemistry, Faculty of Pharmacy, Biruni University, Istanbul, Turkey;; 5 Department of Histology and Embryology, Faculty of Medicine, Biruni University, Istanbul, Turkey;; 6 Department of Basic Medical Sciences, Faculty of Medicine, Biruni University, Istanbul, Turkey;

**Keywords:** Bibliometric analysis, ketogenic diet, aging, scopus, WoSCC, VOSviewer

## Abstract

**Background:**

The potential benefits of the ketogenic diet (KD) on ageing are currently receiving increasing attention. Although there are various studies on this subject in the existing literature, there is a lack of systematic review and bibliometric analysis.

**Aims:**

This study aimed to present a bibliometric overview and visualization analysis of the existing studies examining the relationship between KD and ageing, identify trends in this field, and provide a basis for future research and sustainable development goals.

**Materials and Methods:**

This study involved a systematic review of the literature. In this study, Scopus (Elsevier) and Web of Science Core Collection (WoSCC) databases were used for bibliometric analysis. In the study, all articles, reviews, and other types of publications on KD and ageing published between 1995 and 2024 were analysed. Studies covering the years 1995-2025 and including the keywords ‘ketogenic diet OR ketogenic diets OR ketone diet AND aging AND PUBYEAR > 1995 AND PUBYEAR < 2025' in the title were included. The VOSviewer software (VOSviewer v.1.6.10) was utilized to visualize the data. The data obtained were evaluated by bibliometric methods, such as keyword analysis and cluster analysis.

**Results:**

A significant increase in the number of studies on KD and ageing was observed. In the study, when the data obtained from WoSCC and Scopus databases and VOSviewer analysis results were evaluated together, a total of 10,170 scientific documents in the Scopus database and a total of 168 scientific documents were identified in the Web of Science database between 1995-2025 worldwide. The author publishing the most on the subject was found to be Cunnane, S.C. The country contributing the most to the field was found to be the United States of America (USA). The institution that produced the most documents was Harvard Medical School. In a total of 10,170 records, the most preferred type of publication was articles. Nutrients journal was the journal with the highest number of publications. According to the results of keyword analysis, the words “ketogenic diet” and “aging” were the most frequently used and most strongly related words.

**Conclusion:**

The results of this study showed a significant increase in the number of studies investigating the effects of KD on ageing. More high-quality, randomised controlled clinical trials are needed in this field. In particular, there is a lack of studies examining the effects of KD on age-related diseases at the molecular level.

## 1 INTRODUCTION

Nutrition plays a pivotal role as a determinant of health throughout the life cycle. Attentiveness to daily food intake, diet, and lifestyle is essential for sound clinical care. The achievement of healthy aging and the maintenance of immunity can be realized through the appropriate combination of micronutrients (including vitamins C, A, D, E, and K; zinc; folate; calcium; iron; and B vitamins) and macronutrients (proteins, carbohydrates, and fats). A balanced diet, characterized by suitable nutrient composition, can prevent or delay the onset and complications of prevalent chronic diseases, such as diabetes, hypertension, heart disease, and cancer, as well as conditions commonly associated with aging [1].

Clinicians, practitioners, and athletes frequently seek innovative nutritional strategies to enhance health and performance. There is a growing interest in altering substrate availability through dietary manipulation to influence competitive performance [2].

Normal and healthy aging is associated with substantial changes in body composition and organ function. Aging processes lead to an irreversible decline in normal physiological functions, characterized as time-dependent functional decline and the emergence of age-related diseases. Both genetic and environmental factors have been demonstrated to modulate cellular functions, resulting in aging phenotypes, such as cellular senescence, mitochondrial dysfunction, loss of proteostasis, telomere attrition, dysregulated nutrient sensing, stem cell exhaustion, and epigenetic alterations [3]. While human life expectancy has consistently increased over time, this has not been paralleled by a proportional enhancement in health status. To mitigate the resultant medical, economic, and psychological burdens of this disparity, it is imperative to improve health status by delaying both the aging process and the onset of age-related diseases. Consequently, there is growing interest in developing novel therapeutic interventions to ameliorate the effects of aging and associated diseases and extend life expectancy.

The application of exogenous ketogenic supplements has been demonstrated to be an efficacious method for inducing and maintaining a state of healthy nutritional ketosis [4]. In summary, exogenous ketogenic supplements, such as ketone salts and ketone esters, have the potential to mitigate the aging process, delay the onset of age-related diseases, and extend lifespan through the induction of ketosis.

The ketogenic diet (KD) is a low-carbohydrate, high-fat diet that emulates the metabolic state observed during fasting. KD typically comprises a high-fat, adequate-protein (1 gram/kg), and low-carbohydrate diet that induces metabolic changes akin to those associated with fasting [5]. KD can be categorized into three types: standard KD, targeted KD, and cyclical KD. The standard KD entails deriving 75-80% of daily caloric intake from fats, 15-20% from proteins, and 5-10% from carbohydrates. The targeted KD increases carbohydrate intake to meet the elevated energy demands during exercise. The cyclical KD is a periodic approach that permits higher carbohydrate consumption for several days following strict adherence to KD [6]. The health benefits of KD include weight loss, a reduction in insulin resistance, regulation of blood sugar levels, treatment of epilepsy, support for brain health, and treatment of other metabolic disorders [7, 8].

The interest in bibliometric analysis is growing progressively [9]. Bibliometric analysis is extensively employed to investigate developmental trends and key aspects of research through the quantitative examination of publications [10]. To the best of our knowledge, despite the increasing volume of publications on KD within the field of aging, no systematic review or bibliometric analysis has been undertaken. Therefore, the objective of this study was to identify the most contributing authors, institutions, and countries, the key topics, and the most frequently utilized keywords [11], as well as uncover current research trends in KD related to aging and provide guidance for future research endeavors [12].

## 2 MATERIALS AND METHODS

The Scopus database, developed by Elsevier, a global information analytics company, was employed for bibliometric analysis. This database incorporates the features of the U.S. National Library of Medicine's PubMed database. Scopus facilitates accurate and efficient data analysis across a broad spectrum of fields. Within the Scopus database, results can be searched by document type, keyword, subject area, country, publication year, and topic. Due to these capabilities, the Scopus database was utilized for academic purposes, medical literature research, and citation analysis. Additionally, the database offers the functionality to export analysis results as documents [13]. Web of Science was selected as the secondary database for this study due to its extensive coverage of over 12,000 academic journals and its frequent utilization by researchers [14].

### 2.1 Experimental

A comprehensive analysis was conducted on studies pertaining to “aging and ketogenic diets” published between 1995 and 2025. The most recent literature search, performed on January 31, 2025, in Scopus and Web of Science Core Collection (WoSCC) databases, utilized the following query: 'TITLE-ABS-KEY ((ketogenic AND diet) OR (ketogenic AND diets) OR (ketone AND diet)) AND (aging) AND PUBYEAR > 1995 AND PUBYEAR < 2025'. Quantitative and qualitative analyses of the retrieved data were executed using appropriate bibliometric indicators, encompassing factors, such as publication volume, journal source, research field, country of origin, and citation impact over time.

The VOSviewer software, version 1.6.16 (Centre for Science and Technology Studies, Leiden University, Leiden, The Netherlands), was employed to analyze keywords, construct a co-occurrence network, and visualize the data, thereby elucidating research trends and interrelationships. This study was conducted in adherence to the Declaration of Helsinki. As the analysis was based on existing data, ethical committee approval was not required.

## 3 RESULTS

### 3.1 Distribution of Publications on the Effects of KD on Aging Over the Years

In the study, when the data obtained from WoSCC and Scopus databases and VOSviewer analysis results were evaluated together, a total of 10,170 scientific documents in the Scopus database and a total of 168 scientific documents in the Web of Science database were identified between 1995-2025 worldwide.

**Fig. (1):**
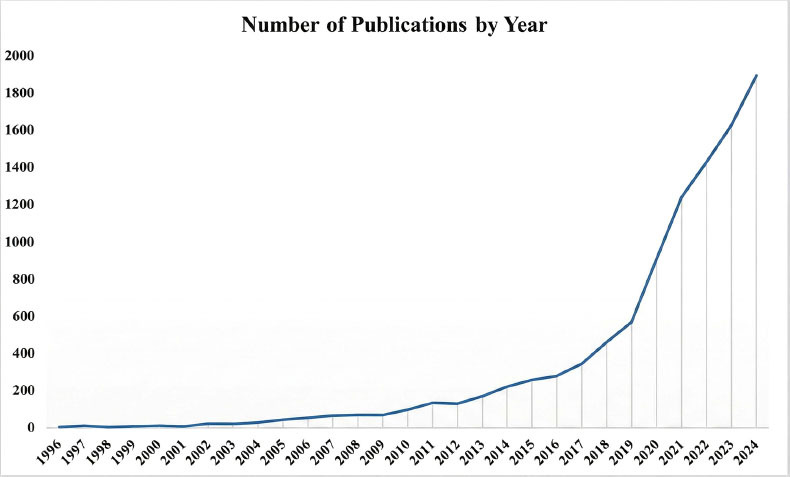
Number of publications worldwide between 1995-2025.

In this study, a total of 10,170 scientific documents were identified in the Scopus database between January 1, 1996, and January 1, 2025, worldwide. According to the analysis, the highest number of publications related to “aging and the ketogenic diet” was observed in 2024 (n = 1.891, 18.60%) (Fig. **1**).

Initially, only 2 publications (0.02%) were recorded in 1996. In 1997, this number increased to 9 publications (0.09%), but it then decreased to 2 publications (0.02%) in 1998. Interest in the topic began to rise in the early 2000s; 20 publications (0.20%) were documented in 2002, followed by 22 publications (0.22%) in 2003. Although this increase was notable relative to 2001, the overall number remained low. In 2004, 28 publications (0.28%) were identified, and by 2006, the number had risen to 54 (0.53%), indicating a gradual increase in research interest regarding the relationship between KD and aging. From 2007 onward, a more pronounced upward trend emerged. In 2007, 65 publications (0.64%) were documented, and similar trends were observed in 2008 and 2009, with 69 (0.68%) and 70 (0.69%) publications, respectively. A more substantial increase was observed in 2010, when the number of publications reached 98 (0.96%). In 2011, 134 publications (1.32%) were recorded, accounting for a rise of approximately 36.73% compared to 2010. This upward trend continued steadily until 2013, amounting to 169 publications (1.66%).

**Fig. (2):**
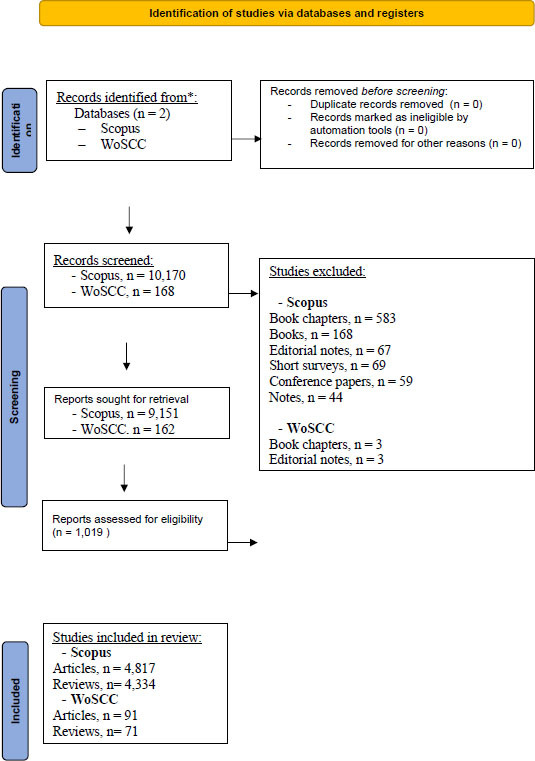
PRISMA 2020 flow diagram for new systematic reviews obtained from Scopus [15]. **Note:** Consider, if feasible to do so, reporting the number of records identified from each database or register searched (rather than the total number across all databases).

In 2014, a marked increase was noted with 222 publications (2.18%), representing a 31.36% increase relative to 2013. This positive trajectory persisted in subsequent years, with 257 publications (2.53%) in 2015 and 280 publications (2.75%) in 2016. In 2017, the output increased to 344 publications (3.38%). The trend accelerated further in 2018 and 2019, yielding 461 publications (4.54%) and 568 publications (5.59%), respectively. A significant surge occurred in 2020, when the number of publications jumped to 910 (8.95%), reflecting an approximately 60.21% increase compared to 2019. This rapid expansion continued in 2021 and 2022, with 1,241 (12.20%) and 1,428 (14.04%) publications, respectively. In 2023, 1,628 publications (16.01%) were identified, and finally, in 2024, the output peaked at 1,891 publications (18.60%), marking the highest level of research interest in this field. (Fig. **2**) illustrates the annual distribution of documents, clearly demonstrating a consistent increase in scholarly attention from 1996 to 2024.

### 3.2 Document Types (Scopus and WoSCC)

In Scopus, the majority of publications were categorized as 'article' (n = 4,814; 47.4%), followed by 'review' (n = 4,333; 42.6%) and other publication types (n = 1,019; 10%). Conversely, in WoSCC, most publications were classified as 'article' (n = 91; 54.167%), followed by 'review article' (n = 71; 42.262%) and other publication types (n = 6; 1.008%). We only selected articles and reviews and eliminated other types of documents, obtaining 9,147 (Scopus) and 162 (WoSCC) documents (Fig. **2**), respectively. Further details on the WoS index are presented in Table **1**.

### 3.3 Top Authors of the Publications on the Effects of KD on Aging

Among the top ten authors with the most publications on the subject, S.C. Cunnane from Canada was found to be the most prolific, having published 57 documents (0.56% of the total) and accumulated 17,354 citations, corresponding to an h-index of 67. Following him, M.P. Mattson from the United States had produced 49 documents (0.48%), amassing an impressive 155,245 citations and achieving the highest h-index in this group at 211. T.N. Seyfried, also from the United States, contributed 38 documents (0.37%), garnering 12,944 citations and an h-index of 63. A. Paoli from Italy had published 30 documents (0.29%), receiving 8,868 citations and attaining an h-index of 44, while G. Muscogiuri, also from Italy, had produced 29 documents (0.29%), with 11,096 citations and an h-index of 59. L. Barrea’s 28 documents (0.28%) had been cited 8,755 times, corresponding to an h-index of 51. M. Fortier from Canada contributed 28 documents (0.28%), with 4,326 citations and an h-index of 26. C. A. Castellano, also from Canada, had published 27 documents (0.27%), accumulating 2,494 citations and an h-index of 27, whereas J. F. Cryan from Ireland, despite an equal publication count of 27 (0.27%), stood out with 85,818 citations and an h-index of 144. Finally, J. M. Rho from the United States had published 26 documents (0.26%), amassing 10,327 citations and achieving an h-index of 53 (Table **2**).

### 3.4 Top 10 Journals with the Most Publications on the Effects of KD on Aging

The top 10 journals with the most publications on “aging and the ketogenic diet”, along with their Scimago Journal Rank (SJR), CiteScore, and Source Normalized Impact per Paper (SNIP) values, are presented in Table **2**. Collectively, these journals accounted for 1,491 (14.66%) of all identified documents. According to the data, Nutrients had the highest number of publications, accounting for a total of 499 (4.91%). Its SJR was 1.301, CiteScore was 9.2, and SNIP was 1.306. The International Journal of Molecular Sciences ranked second, featuring 323 documents (3.18%), with an SJR of 1.179, a CiteScore of 8.1, and an SNIP of 1.120. The third most common journal was Frontiers in Nutrition, which published 141 documents (1.39%), with an SJR of 0.828, a CiteScore of 5.2, and an SNIP of 0.878 (Table **3**).

Other prominent journals included PLOS One, with 95 documents (0.93%) (SJR: 0.839, CiteScore: 6.2, SNIP: 1084.0), and Scientific Reports, with 93 documents (0.91%) (SJR: 0.900, CiteScore: 7.5, SNIP: 1.182). Antioxidants followed with 73 documents (0.72%) (SJR: 1.222, CiteScore: 10.6, SNIP: 1.381), while Frontiers in Neuroscience included 71 documents (0.70%) (SJR: 1.063, CiteScore: 6.2, SNIP: 0.988). Frontiers in Aging Neuroscience featured 69 documents (0.68%) (SJR: 1173.0, CiteScore: 6.3, SNIP: 0.900), and Ageing Research Reviews contributed 67 documents (0.66%) (SJR: 3.376, CiteScore: 19.8, SNIP: 2.620). Finally, Frontiers in Endocrinology published 60 documents (0.59%) (SJR: 1.240, CiteScore: 5.7, SNIP: 1.122) (Table **3**).

### 3.5 Institutions with the Most Publications on the Effects of KD on Aging

Among the leading institutions, the Ministry of Education of the People's Republic of China led as the institution with the most publications, totaling 196 documents (1.93%). Harvard Medical School in the United States ranked second with 169 documents (1.66%), followed by Inserm (Institut National de la Santé et de la Recherche Médicale) in France with 144 documents (1.42%). Sapienza Università di Roma in Italy held fourth place, with 112 documents (1.10%) (Table **4**).

In fifth place was the Johns Hopkins University School of Medicine (United States), with 107 documents (1.05%), closely followed by the National Institutes of Health (NIH), also in the United States, with 106 documents (1.04%). The University of California, San Francisco (US), stood in seventh place with 105 documents (1.03%). The University of Toronto in Canada ranked eighth, having contributed 97 documents (0.95%). Rounding out the top ten were the University of Alabama at Birmingham (US), with 92 documents (0.90%), and the University of South Florida, Tampa (US), with 91 documents (0.89%). Table **3** lists the top ten institutions contributing the most documents to the literature on this subject, along with their respective countries and affiliation IDs (Table **4**).

### 3.6 Ten Countries with the Most Publications of Papers/ Documents on the Ketogenic Diet's Relationship with Aging

The number of publications on aging and KD by country is shown in Table **5**. Using VOSviewer (v.1.6.10), the data were analyzed based on a minimum of four publications and four citations per country. When the distribution of these records by country was examined, the United States of America (USA) emerged as the leading contributor, with 3,475 documents (34.17%). China ranked second with 1,931 documents (18.99%), followed by Italy in third place with 858 documents (8.44%). The United Kingdom came fourth with 739 documents (7.27%), and Germany was fifth with 542 documents (5.33%). Canada ranked sixth with 527 documents (5.18%), while Spain contributed 448 documents (4.41%) and Australia 433 documents (4.26%). India occupied ninth place with 355 documents (3.49%), and France completed the top ten with 328 documents (3.23%) (Table **5**).

For cluster analysis of 52 keywords, VOSviewer was used. The frequency of use of the keywords, their relationship with each other, and the topics of the clusters were analyzed. Frames and labels were used to form an element and colors distinguished different clusters from each other. Fig. (**3**) shows that the keywords formed 4 clusters, indicating at least 4 research directions. The green cluster represents the largest cluster classified as a regulatory mechanism. The large circles indicate keywords that appeared with high frequency, and the lines between the circles indicate the presence of a link between keywords. The distance between two terms indicates the closeness of the relationship (the shorter the line, the stronger the relationship).

### 3.7 Sustainable Development Goals

The data from WoSCC were analyzed for the occurrence of 'aging and KD' in research studies. The analysis revealed that 'Good Health and Well-Being' accounted for a significant portion of the research studies in this context, with 160 studies (95.238%). 'Zero Hunger' ranked second with 2 studies (1.19%), while 'Gender Equality' ranked third, also with 2 studies (1.19%).

## 4 DISCUSSİON

Various electronic databases can be utilized for bibliometric studies. Elsevier developed Scopus, which provides more comprehensive and accurate data analysis than PubMed or Google Scholar, by combining features of both Web of Science and PubMed [15]. Scopus allows for exporting documents and initiating new searches from categorized results based on document type, source title, author name, year of publication, and subject area [16-18].

Aging is characterized as a multifactorial process encompassing a broad range of physiological effects, including the onset of age-related diseases and eventual mortality. Although recent years have witnessed an increase in the number of anti-aging interventions, the emergence of distinctive features of aging, particularly restrictive diets, such as calorie restriction (CR) and intermittent fasting (IF) strategies, have gained popularity. IF exerts its effects through the activation of bioenergetic sensors and longevity-associated genes, such as AMPK and sirtuins. Among the health benefits of fasting are reductions in body weight and obesity, as well as a decreased incidence of diseases, such as cancer, cardiovascular disorders, neurodegeneration, inflammation, and metabolic syndrome. KD and Mediterranean diet are among the variants that are widely embraced as IF regimens [19].

Numerous studies examining the impact of KD on aging have suggested potential anti-aging benefits associated with this dietary regimen. Research has demonstrated that KD can enhance mitochondrial function, mitigate inflammation, and elevate the production of antioxidants, all of which may contribute to healthy aging. Additionally, KD may exert neuroprotective effects. As cognitive decline becomes more prevalent with age, certain studies have indicated that KD can enhance cognitive function in older individuals [20]. However, some research has reported that KD does not significantly affect longevity and is associated with an increased risk of cardiovascular disease, cancer, and diabetes [21].

Sarcopenia, characterized by a decline in both the number and functional capacity of muscle fibers, is a prevalent age-related condition. It affects approximately 14% of individuals aged 65-69 years and escalates to 50% in those aged 80 years and above. Sarcopenia significantly impairs the ability to perform activities of daily living, leading to immobility, reduced flexibility, and an increased susceptibility to morbidity and mortality. Age-related muscle atrophy, manifested by a decrease in muscle fiber count and diameter, is a primary contributor to this condition [22, 23]. Calorie restriction is recognized as a potent intervention for mitigating age-related decline, and KD is posited as a potential mechanism through which muscle mass preservation is achieved in older adults [24, 25].

Adherence to a KD confers a spectrum of health benefits, encompassing neuroprotection, enhanced mitochondrial function, autophagy activation, and anti-inflammatory effects [26-28]. In a study by Wallace *et al*., the skeletal muscle of mice fed KD for 14 months was analyzed. Compared to the normal control group, the KD-fed mice exhibited a significant increase in leg muscle mass, upregulation of mitochondrial gene expression, enhanced metabolic oxidation, and improved antioxidant capacity. Furthermore, the KD regimen led to a reduction in endoplasmic reticulum stress and protein synthesis [29]. These alterations may contribute to a favorable cellular milieu, mitigate oxidative stress, and consequently, facilitate the maintenance of muscle fiber number and functionality, thereby attenuating age-related decline. Regarding lifespan extension, β-hydroxybutyrate (β-HB) has been implicated in promoting longevity in worms through dual anti-aging pathways. Specifically, β-HB inhibits histone deacetylases (HDACs), resulting in increased DAF-16/FOXO activity, and modulates mitochondrial metabolism. Additionally, β-HB and SKN-1/Nrf2 activate the antioxidant response pathway [30]. Moreover, KD has been demonstrated to enhance lifespan and survival in mice by augmenting protein acetylation and diminishing tissue-specific mTOR complex 1 activation [31].

A study examining the hypoalgesic and anti-inflammatory effects of KD demonstrated that ad libitum KD feeding in juvenile and adult rats resulted in reduced nociception, as evidenced by increased hind paw withdrawal latency, and diminished peripheral inflammation, as indicated by decreased complete Freund's adjuvant (CFA)-induced hind paw edema and plasma extravasation. To date, clinical applications of ketogenic interventions have predominantly centered on the established efficacy in pediatric epilepsy [32] and diabetes management [33]. However, recent translational research is expanding the scope of clinical outcomes to encompass brain cancer, traumatic brain injury, and Rett syndrome [34-36]. Given the pressing need for novel therapies targeting pain, inflammation, and inflammatory pain, current data suggest that further translational research is warranted to explore the therapeutic potential of KD and related metabolic interventions.

In a 12-month randomized controlled trial conducted by Saslow *et al*., 34 overweight and obese participants were assigned to either a low-carbohydrate KD or a low-fat, moderate-carbohydrate diet. The KD group demonstrated a significantly greater reduction in hemoglobin A1c (HbA1c) level, a marker of long-term blood glucose control, compared to the low-fat, moderate-carbohydrate group. Furthermore, the study suggested that KD may be effective in reducing the need for diabetes medication [37].

In a study conducted by McKenzie *et al*., 262 patients with diabetes were put on KD with physician-supervised medication adjustments. After 70 days, assessments revealed consistent carbohydrate restriction, as indicated by elevated mean beta-hydroxybutyrate levels, a reduction in glycosylated hemoglobin levels, and a decrease in diabetes medication usage. Furthermore, patients with diabetes who adhered to KD for one year demonstrated improvements in cardiovascular disease risk markers, lipid profiles, LDL particle size, blood pressure, and inflammatory markers compared to those following a standard diet [38]. Hallberg *et al*. reported that participants adhering to KD experienced a reduction in glycosylated hemoglobin levels. Furthermore, the study observed a decrease in diabetes medication usage, with 40% of participants able to discontinue exogenous insulin and 60% able to halve their daily insulin dosage [39]. Furthermore, a substantial body of research examining ketogenic and non-ketogenic low-carbohydrate diets in individuals with type 2 diabetes has concluded that insulin sensitivity improves in response to lower glycosylated hemoglobin values and reduced carbohydrate intake [40]. While the majority of studies have focused on patients with type 2 diabetes, there is no inherent contraindication for low-carbohydrate dietary therapy in individuals with type 1 diabetes. Leow *et al*. demonstrated that adults with type 1 diabetes who adhered to KD for 30 months exhibited a significant reduction in glycosylated hemoglobin levels while maintaining euglycemia [41].

While KD demonstrates potential anti-aging properties, it is essential to contextualize these findings relative to other dietary regimens, such as the Mediterranean diet (MD), intermittent fasting (IF), caloric restriction (CR), and plant-based diets. Each of these dietary strategies exerts distinct metabolic effects and may offer unique benefits for aging populations.

### 4.1 Mediterranean Diet (MD)

Unlike KD, which primarily shifts metabolism toward ketone utilization, MD emphasizes a balanced intake of macronutrients with an abundance of polyphenols, omega-3 fatty acids, and fiber. MD is strongly associated with cardiovascular health, reduced inflammation, and improved cognitive function. The absence of extreme macronutrient restriction makes it a sustainable option for long-term adherence, particularly in aging populations at risk of metabolic disorders [42, 43].

### 4.2 Intermittent Fasting (IF)

IF shares mechanistic similarities with KD, particularly in promoting autophagy and mitochondrial resilience. However, while KD relies on chronic carbohydrate restriction to sustain ketosis, IF achieves similar metabolic benefits through cyclic fasting periods. IF has been shown to improve insulin sensitivity, reduce oxidative stress, and enhance longevity through the activation of sirtuins and AMPK signaling [44, 45].

### 4.3 Caloric Restriction (CR)

It is one of the most extensively studied dietary interventions for lifespan extension. It has been demonstrated to delay aging processes in multiple model organisms through reduced oxidative stress, enhanced DNA repair, and increased autophagy. Unlike KD, which allows for ad libitum intake of fats, CR imposes a consistent energy deficit, requiring careful nutrient planning to prevent deficiencies in aging individuals [46-49].

### 4.4 Plant-based Diets

Diets rich in plant-based foods, such as vegetarian and vegan diets, are associated with reduced inflammation, improved gut microbiota composition, and lower risks of chronic diseases. While these diets do not induce ketosis, they offer protective effects against metabolic and cardiovascular disorders, making them viable alternatives to KD for aging populations who may struggle with high-fat intake [49-51].

### 4.5 Potential Risks and Controversies in KD Research

Although KD has been associated with numerous metabolic benefits, including improved cognitive function, insulin sensitivity, and anti-inflammatory effects, concerns persist regarding its long-term safety and broader applicability, particularly in aging populations. One of the primary controversies surrounding KD is its impact on cardiovascular health [52, 53]. While some studies indicate that KD leads to improved lipid profiles by increasing high-density lipoprotein (HDL) levels and shifting low-density lipoprotein (LDL) particle size toward a less atherogenic profile, other studies have reported elevated LDL cholesterol and triglyceride levels, raising concerns about potential atherosclerotic risks. The discrepancy in findings suggests that individual genetic and metabolic factors may play a crucial role in determining the cardiovascular outcomes of KD [54, 55].

Furthermore, concerns have been raised regarding the long-term impact of KD on muscle preservation and physical performance. Although KD has been shown to promote fat oxidation and metabolic flexibility, some studies suggest that carbohydrate restriction may impair muscle protein synthesis and reduce exercise performance, particularly in high-intensity anaerobic activities. This is particularly relevant for aging populations, as muscle loss (sarcopenia) is a significant contributor to frailty and reduced quality of life. Incorporating adequate protein intake and resistance training may help mitigate these effects, but further research is required to establish optimal dietary protocols [56].

Another widely debated aspect of KD is its potential impact on bone health. Several studies have reported that long-term adherence to KD may lead to reduced bone mineral density, possibly due to alterations in calcium metabolism and increased acid load from high dietary fat consumption. This raises concerns, particularly for aging individuals who are already at higher risk for osteoporosis and fractures. Further research is needed to determine whether these risks can be mitigated through dietary modifications or supplementation [57].

The effects of KD on kidney function have also been scrutinized. Due to its high protein and fat content, KD may increase the renal acid load, potentially exacerbating kidney disease in susceptible individuals [58]. Some evidence suggests that chronic ketosis may lead to an increased risk of nephrolithiasis (kidney stones), possibly due to altered citrate and calcium excretion [59]. While these effects appear to be more pronounced in individuals with pre-existing renal conditions, they highlight the need for careful monitoring and personalized dietary recommendations.

Lastly, the psychological and behavioral aspects of KD warrant consideration. Adherence to KD can be challenging due to its restrictive nature, potentially leading to increased rates of diet discontinuation, nutrient deficiencies, and disordered eating behaviors [60]. Additionally, prolonged ketosis may alter gut microbiota composition, with potential implications for immune function and metabolic health [61]. A more comprehensive understanding of these effects is necessary to determine the long-term sustainability of KD as a dietary intervention for aging populations.

### 4.6 Future Directions

To advance our understanding of KD in the context of aging, future research should adopt a multi-faceted approach that integrates diverse methodologies and perspectives. The following key areas should be prioritized:

• **Integrative Omics Approaches**: The application of metabolomics, transcriptomics, epigenomics, and proteomics will provide a more comprehensive understanding of the molecular mechanisms underlying KD’s effects on aging. These approaches will help identify potential biomarkers that can predict individual responses to KD and its long-term impact on health.

• **Longitudinal Clinical Trials**: Well-designed, long-term randomized controlled trials (RCTs) are essential to evaluate the sustained effects of KD on aging-related health outcomes, including cognitive function, cardiovascular health, metabolic regulation, and musculoskeletal integrity. These studies should include diverse populations to ensure generalizability.

• **Personalized Nutrition Strategies**: Given the variability in metabolic and genetic responses to KD, future research should focus on precision nutrition approaches that tailor KD interventions based on individual risk factors, genetic predispositions, and metabolic profiles. This will enhance both efficacy and safety.

• **Comparative Dietary Analyses**: While KD has demonstrated potential benefits, its long-term effects must be evaluated in comparison to other dietary regimens, such as intermittent fasting, caloric restriction, and plant-based diets. Investigating the relative advantages and disadvantages of these dietary approaches in aging populations will provide valuable insights for clinical recommendations.

• **Global Research Collaborations**: Expanding KD research to underrepresented regions will improve our understanding of its applicability across different ethnic and socio-economic populations. Multinational studies will help uncover cultural, environmental, and genetic factors that influence KD’s effects on aging.

• **Nutrient Optimization and Hybrid Approaches**: Research should explore modified KD versions, such as the inclusion of plant-based fats, fiber-enriched formulations, or cyclic KD approaches, to enhance sustainability and minimize potential risks. Additionally, investigating KD’s interaction with exercise and pharmacological interventions could provide more holistic strategies for aging-related health optimization.

By addressing these critical areas, future research will pave the way for more personalized, sustainable, and effective dietary interventions that optimize aging health while minimizing potential risks associated with KD.

## CONCLUSION

The aforementioned KD administration usually starts with a short fasting period, and changes in plasma ketones, insulin, glucose, glucagon, and free fatty acids may occur within hours of starting the diet; this fasting period may be quite deep. However, it should be taken into consideration that KD may also cause problems related to high-fat consumption in the long term, which may also have potential side effects. With the bibliometric analysis we have conducted, the current research trends regarding the effects of KD on aging have been evaluated extensively, aiming to guide future research.

## Figures and Tables

**Fig. (3):**
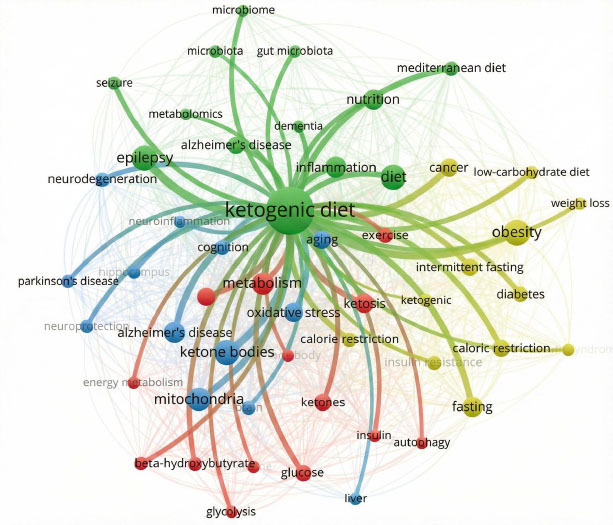
Keyword network analysis of publications.

**Table 1:** Web of Science index.

Web of Science Index	Documents	%
Science Citation Index Expanded (SCI-EXPANDED)	127	75.59
Social Sciences Citation Index (SSCI)	18	10.71
Emerging Sources Citation Index (ESCI)	13	7.73
Book Citation Index – Science (BKCI-S)	7	4.16
Conference Proceedings Citation Index – Science (CPCI-S)	3	1.78

**Table 2:** Top 10 most active authors (1995-2025).

Author	Documents	%	Total Number of Citations (S)	h-index (S)	Country
Cunnane, S.C.	57	0.56047	17,354	67	Canada
Mattson, M.P.	49	0.48181	155,245	211	US
Seyfried, T.N.	38	0.37365	12,944	63	US
Paoli, A.	30	0.29499	8,868	44	Italy
Muscogiuri, G.	29	0.28515	11,096	59	Italy
Barrea, L.	28	0.27532	8,755	51	Italy
Fortier, M.	28	0.27532	4,326	26	Canada
Castellano, C.A.	27	0.26549	2,494	27	Canada
Cryan, J.F.	27	0.26549	85,818	144	Ireland
Rho, J.M.	26	0.25565	10,327	53	US

**Note:** %: The ratio of the total number of articles. These percentages were calculated by dividing Documents number to 10,170.

**Table 3:** Top 10 journals with the highest publication count (1995-2025).

Source	Documents	%	SJR	CiteScore	SNIP
Nutrients	499	4.91	1.301	9.2	1.306
International Journal of Molecular Sciences	323	3.18	1.179	8.1	1.120
Frontiers in Nutrition	141	1.39	0.828	5.2	0.878
Plos One	95	0.93	0.839	6.2	1.084
Scientific Reports	93	0.91	0.900	7.5	1.182
Antioxidants	73	0.72	1.222	10.6	1.381
Frontiers in Neuroscience	71	0.70	1.063	6.2	0.988
Frontiers in Aging Neuroscience	69	0.68	1.173	6.3	0.900
Ageing Research Reviews	67	0.66	3.376	19.8	2.620
Frontiers in Endocrinology	60	0.59	1.240	5.7	1.122

**Abbreviations:** Scimago Journal Rank (SJR), Source Normalized Impact per Paper (SNIP)

**Table 4:** The ten organisations providing the most documents to the literature on the subject.

Affiliation	Country	Affiliation ID	Documents	%
Ministry of Education of the People's Republic of China	China	60001604	196	1.927
Harvard Medical School	US	60002746	169	1.661
Inserm	France	60000905	144	1.415
Sapienza Università di Roma	Italy	60032350	112	1.101
Johns Hopkins University School of Medicine	US	60001117	107	1.052
National Institutes of Health NIH	US	60006577	106	1.042
University of California, San Francisco	US	60023691	105	1.032
University of Toronto	Canada	60016849	97	0.953
The University of Alabama at Birmingham	US	60027086	92	0.904
University of South Florida, Tampa	US	60007740	91	0.894

**Table 5:** Distribution of the number of publications by country.

Country/Territory	Documents	%
United States	3475	34.1691
China	1931	18.987
Italy	858	8.436
United Kingdom	739	7.266
Germany	542	5.329
Canada	527	5.181
Spain	448	4.405
Australia	433	4.257
India	355	3.490
France	328	3.225

## Data Availability

All data generated or analyzed during this study are included in this published article.

## LIST OF ABBREVIATIONS

WoSCC: Web of Science Core Collection
KD: Ketogenic Diet
CR: Calorie Restriction
IF: Intermittent Fasting
CFA: Complete Freund's Adjuvant
HbA1c: Hemoglobin A1c
β-HB: β-hydroxybutyrate
HDACs: Histone Deacetylases
MD: Mediterranean Diet
RCTs: Randomized Controlled Trials
HDL: High-Density Lipoprotein
LDL: Low-Density Lipoprotein

## STANDARDS OF REPORTING

PRISMA guidelines were followed.
